# Robust AAV Genotyping Based on Genetic Distances in Rep Gene That Are Maintained by Ubiquitous Recombination

**DOI:** 10.3390/v14051038

**Published:** 2022-05-13

**Authors:** Marina I. Beloukhova, Alexander N. Lukashev, Pavel Y. Volchkov, Andrey A. Zamyatnin, Andrei A. Deviatkin

**Affiliations:** 1 Institute of Molecular Medicine, Sechenov First Moscow State Medical University, 119991 Moscow, Russia; zamyat@belozersky.msu.ru; 2 Martsinovsky Institute of Medical Parasitology, Tropical and Vector-Borne Diseases, First Moscow State Medical University (Sechenov University), 119991 Moscow, Russia; alexander_lukashev@hotmail.com; 3 Genome Engineering Lab, Moscow Institute of Physics and Technology (National Research University), 141700 Dolgoprudniy, Russia; vpwwww@gmail.com; 4 The National Medical Research Center for Endocrinology, 117036 Moscow, Russia; 5 Belozersky Institute of Physico-Chemical Biology, Lomonosov Moscow State University, 119992 Moscow, Russia; 6 Department of Biotechnology, Sirius University of Science and Technology, 1 Olympic Ave, 354340 Sochi, Russia; 7 Department of Immunology, Faculty of Health and Medical Sciences, University of Surrey, Guildford GU2 7XH, UK; 8 Laboratory of Postgenomic Technologies, Izmerov Research Institute of Occupational Health, 105275 Moscow, Russia

**Keywords:** adeno-associated virus, AAV, classification, genotype, serotype, intraspecies, pairwise genetic distance, recombination

## Abstract

Adeno-associated viruses (AAVs) are a convenient tool for gene therapy delivery. According to the current classification, they are divided into the species AAV A and AAV B within the genus Dependoparvovirus. Historically AAVs were also subdivided on the intraspecies level into 13 serotypes, which differ in tissue tropism and targeted gene delivery capacity. Serotype, however, is not a universal taxonomic category, and their assignment is not always robust. Cross-reactivity has been shown, indicating that classification could not rely on the results of serological tests alone. Moreover, since the isolation of AAV4, all subsequent AAVs were subdivided into serotypes based primarily on genetic differences and phylogenetic reconstructions. An increased interest in the use of AAV as a gene delivery tool justifies the need to improve the existing classification. Here, we suggest genotype-based AAV classification below the species level based on the *rep* gene. A robust threshold was established as 10% nt differences within the 1248 nt genome fragment, with 4 distinct AAV genotypes identified. This distinct sub-species structure is maintained by ubiquitous recombination within, but not between, *rep* genes of the suggested genotypes.

## 1. Introduction

### 1.1. AAV Biology

Adeno-associated viruses (AAVs) are non-enveloped particles with a size of 18–26 nm. Sixty protein molecules form an icosahedral capsid. The genome is represented by a linear single-stranded DNA of approximately 4.7 thousand bases.

The AAV genome contains two open reading frames (ORFs): (1) *rep*, which encodes four overlapping proteins necessary for the regulation of viral DNA replication: Rep78, Rep68, Rep52, and Rep40 [1,2]; and (2) *cap* encoding viral capsid proteins: VP1, VP2 and VP3 [3,4]. The genome is flanked by inverted terminal repeats (ITRs) [5], which form a T-shaped self-complementary secondary structure with a free 3′-hydroxyl that acts as a replication primer [6,7].

For effective replication and reproduction in host cells, AAV requires co-infection with an auxiliary virus, e.g., adenovirus (hence the name adeno-associated), although AAV replication is also possible with herpesvirus, cytomegalovirus, and papillomavirus co-infection [8].

The best-known hosts of AAVs are primates and humans [9,10], although these viruses have been found in other animals [11,12,13]. AAVs do not cause a significant immune response or any notable pathology in the host cells. Consequently, they are not considered pathogenic [4]. Notably, over 90% of the adult population is seropositive to AAVs, i.e., are likely asymptomatic carriers [14]. The prevalence of AAV in distinct tissues varies between 37 and 72% [15].

### 1.2. AAV Gene Therapy

AAVs are a convenient tool for gene therapy [16,17]. Since wild AAVs persist in the form of episomes (integration into the host genome at AAVS1 19q13.3-qter site is extremely rare [18] and absent in vectors), they are safer compared to retroviruses, which insert into the host genome randomly, leading to oncogenesis [19]. AAVs also have reduced immunogenicity compared to adenoviruses, further supporting their utility as gene therapy vectors [20].

Results of preclinical and clinical studies have demonstrated that AAV vectors are safe and effective tools in gene therapy for a range of diseases including cystic fibrosis, hemophilia B, arthritis, hereditary emphysema, muscular dystrophy, Parkinson’s disease, Alzheimer’s disease, prostate cancer, malignant melanoma, epilepsy, and others [16,21,22,23]. To date, three AAV-vector-based products have been approved for use in medical practice: Glybera (familial lipoprotein lipase deficiency) [24], Luxturna (hereditary retinal dystrophy) [25] and Zolgensma (spinal muscular atrophy) [26].

### 1.3. AAV Classification

Current AAV classification is based on the phenotype (the shape of the virion), replication peculiarities, and the host range [27]. According to these criteria, AAVs are represented by two species—AAV A and AAV B. Besides AAVs, there are eight other species within the genus dependovirus (ICTV: https://talk.ictvonline.org/taxonomy/ accessed 25 June 2021).

Additionally, AAVs are subdivided on the intraspecies level into serotypes. The first reports of serologically distinct AAVs date back to 1966, when the first three serotypes were identified [28].

Further, any newly identified AAV serologically distinct from known types were assigned a new serotype in chronological order. To date, 13 AAV serotypes are known. However, there are significant ambiguities and controversies in the properties and definitions of the AAV serotypes. For instance, AAV4 was identified based on the reaction with antiserum [29], whereas AAV5 was assigned based on the DNA structure differences identified by restriction analysis and blot-hybridization [30,31]. This protocol could correspond to the identification of a new genotype, which is consistent with the later assignment of AAV5 to a separate species within the dependovirus genus by the International Committee on Taxonomy of Viruses [32].

AAV6 was assigned a new serotype based on genetic differences from the complete genomes of AAV2 and AAV3 (82% identity), as well as AAV4 (75–78% identity) [33]. However, its cap genes were 96% similar to AAV1, and most likely AAV6 is a variant of AAV1. Despite this, AAV serotype 6 is still in use.

In 2002, during an investigation of the asymptomatic presence of AAV in primate tissues, Gao et al. identified AAV7 and AAV8 [34]. The authors used signature regions—a fragment of genomic sequence at positions 2886–3143 nt unique to each AAV type (previously identified by Rutledge et al. [33])—to define distinct virus types, and named the newly isolated viruses according to the differences in these regions. The *rep* and *cap* nucleotide sequences and the predicted amino acid sequence comparisons for AAV1-8 (with the exception of AAV5 as obviously incongruent) revealed differences, primarily in the region encoding capsid proteins. AAV7 was shown to have a 63% to 85% similarity to the amino acid sequences and 68% to 84% of the nucleotide sequences of other AAV serotypes; similar results were obtained for AAV8. The serological difference of AAV7, AAV8, and other serotypes was also established based on the absence of neutralization by any antiserum other than their own (anti-AAV7 and anti-AAV8, respectively).

Later, Gao et al. identified 11 phylogenetic groups based on phylogenetic analysis of cap sequences—so-called ‘clades’—from A to F, consisting of phylogenetically similar representatives from three or more sources, and five groups of clones (phylogenetically similar representatives from less than three sources) [35].

AAV10 and AAV11 were also identified based on the signature region differences and characterized according to the cap sequence [36]. The serological analysis confirmed that AAV10 and AAV11 were serologically distinct from AAV2.

Similarly, AAV12 was identified based on the *rep* and *cap* sequence differences [37]. Finally, AAV(VR-942) was isolated in 2008 and had a high degree of amino acid sequence similarity of rep with AAV4 (98%) and AAV3 (93%), as well as 93% identity in VP1 as compared to AAV3. Despite this, AAV(VR-942) has a distinct pattern of cellular receptor interaction and was suggested as a new serotype, AAV13 (the name is currently present only in the corresponding GenBank entry); it should be noted that serological studies were not conducted for AAV13 [38].

Thus, since the isolation of AAV4, all subsequent AAVs were subdivided into different serotypes based primarily on genetic differences and phylogenetic reconstructions. In addition to the established 13 serotypes, one of which comprises a separate virus species (AAV5), more than 100 novel genetic variants (so-called serovars [39]) are not assigned to any taxonomic unit below the species level. It is unlikely that they will be all tested serologically, and the genetic boundaries of types are not robust.

Here, we analyzed all available AAV-A sequences to test the possibility of systematically distinguishing AAVs according to genomic sequence data and to suggest a classification based on genotypes with a measurable robust threshold.

## 2. Materials and Methods

### 2.1. Sequence Selection for Analysis

All sequences with a length limited to 4000–5100 nt, which approximately corresponds to the full genome, available as of June, 2021, for Dependoparvovirus isolates were retrieved from the GenBank database (artificial sequences were excluded). Since this study aimed to characterize genetic relationships at the intraspecies level, only sequences of the species AAV A were selected. For this purpose, a phylogenetic tree for the retrieved sequences was constructed using neighbor-joining, implemented in MEGA [40].

### 2.2. Recombination Analysis

The selected sequences were aligned using MAFFT online [41]. Multiple recombination events were analyzed by the RDP4 software [42] (RDP4 output is available at https://github.com/AndreiDeviatkin/repo/blob/main/AAV.rdp, accessed on 11 May 2022). A more comprehensive recombination screening was carried out using pairwise distance deviation matrices (PDDM) and pairwise distance correspondence plots (PDCP) as described earlier [43] with an online Shiny web application (https://v-julia.shinyapps.io/recdplot_app/, accessed on 25 June 2021). Similarity plots for selected sequences were generated using Simplot 3.5.1 [44].

### 2.3. Nucleotide and Predicted Amino Acid Sequences Analysis

The analysis of nucleotide and inferred amino acid sequence differences was carried out in R software.

## 3. Results

### 3.1. The Dataset

A total of 272 Dependoparvovirus sequences (4000–5100 nt length) were retrieved from GenBank. Of them, 105 sequences belonged to the AAV A species based on phylogenetic analysis (Figure S1). The dataset included all previously characterized genotypes, with the exception of AAV9, for which only VP1 coding sequence is available, as well as other unclassified variants available in the database. Two sequences (MK163941.1 and MK163942.1) were significantly different from the others and thus excluded from the dataset.

### 3.2. Recombination Analysis

**Figure 1:**
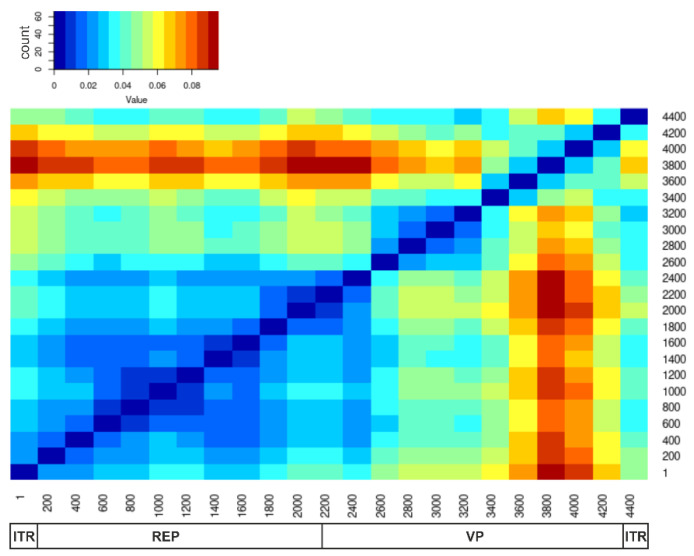
Pairwise distance deviation matrix (PDDM) for AAV A alignment. PDDM summarizes the multitude of pairwise nucleotide distance comparison plots (PDCP) for all possible genome region pairs, visualized as a heatmap. A higher (“red”) value of the color key indicates lower overall sequence distance congruence between two genome regions, which is likely caused by recombination.

Recombination occurs more frequently in some fragments of the virus genome than in others. The root mean square error (RMSE) of all pairwise distances for two genomic regions from the regression line reflects the extent of phylogenetic inconsistency between these fragments of the genome. A high RMSE (indicated by red in Figure 1) indicates more frequent recombination between the corresponding regions of the genome. A pairwise distance deviation matrix (PDDM) was built for all possible pairs of genomic regions using a sliding window (500 nt). Based on the PDDM, a region covering genome positions 200–1700 nt was identified as the region with the lowest relative incidence of recombination, which approximately corresponds to the coding sequence of Rep proteins (Figure 1).

**Figure 2:**
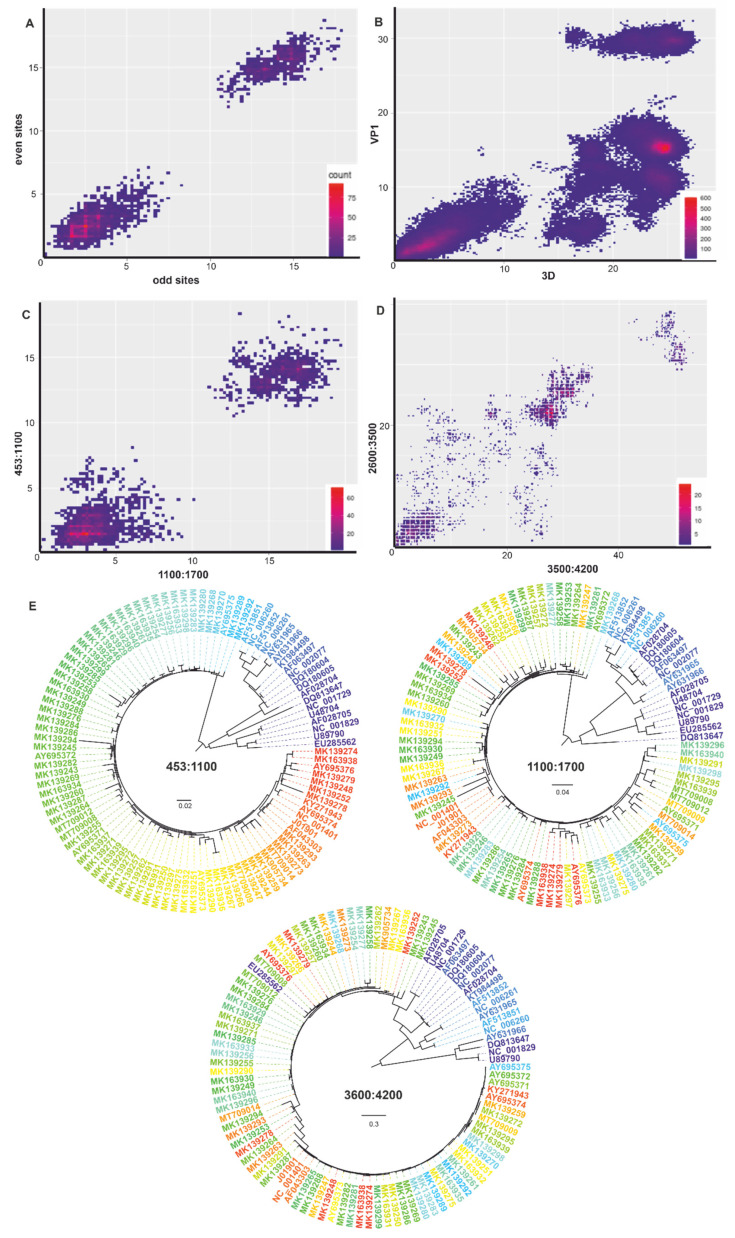
Pairwise nucleotide distance comparison plots (PDCP) indicate recombination between two genetic regions. Each dot corresponds to a pair of nucleotide distances between the same pair of genomes in two genomic regions (axis labels): (**A**) PDCP of even and odd sites in the 453–1700 nt fragment of AAV A alignment (negative control); (**B**) PDCP between VP1 and 3D regions of Enterovirus A species [45], a pattern typical to ubiquitous recombination (positive control); (**C**) PDCP between positions 453 to 1100 nt and 1100 to 1700 of AAV A alignment; (**D**) pairwise distance plot between positions 2600 to 3500 nt and 3500 to 4200 of AAV A alignment. The color indicates the density of overlapping values; (**E**) Phylogenetic trees for three regions in the AAV genome (453–1100; 1100–1700; 3600–4200). The colors of sequences match in trees for each genome region.

The PDCP and phylogenetic reconstruction confirmed that recombination in this genome region occurred only between closely related viruses that differed by less than 10% in their nucleotide sequence (Figure 2C, compare to control Figure 2A). Despite ubiquitous recombination within four phylogenetic clades formed by such closely related viruses, there were no signs of recombination between them (Figure 2E). This pattern contrasted with recombination both within and between these four clades when comparing rep and cap genes on the phylogenetic trees (Figure 2E) and signs of recombination between viruses that differed by over 20% nucleotide sequences in cap (Figure 2D). Since complete genomic sequences were not available for all known AAV types (for AAV10-13 only rep + cap complete coding sequences were accessible), alignment positions 453–1700 (genome positions 470–1700 in the reference sequence #NC_001401) were selected for further analysis. Therefore, the region of rep between positions 453 and 1700 was indeed devoid of long-distance recombination and was thus used for further analysis.

### 3.3. Nucleotide and Amino Acid Pairwise Distance Analysis

**Figure 3:**
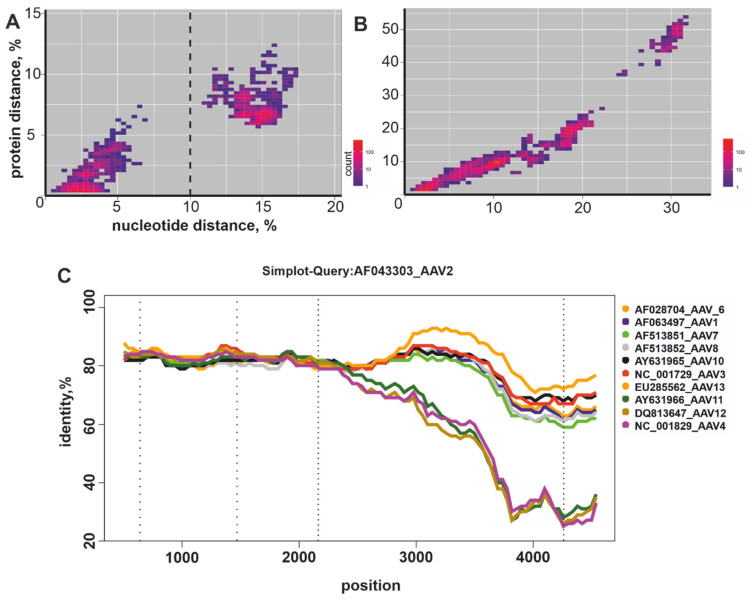
Pairwise amino acid and nucleotide distance plot of AAV A representatives (*n* = 103): (**A**) for 453–1700 nt region; (**B**) for 2250–4400 nt region. Each dot corresponds to amino acid and nucleotide distances of one virus pair. (**C**) Similarity plot demonstrate mosaic genome patterns in AAV. Similarity scan was conducted using alignments of eleven nucleotide sequences indicated in the legend with window/step size 1000/40 nt. Dotted lines indicate 453–1700 nt and 2250–4400 nt regions.

Nucleotide or protein sequences may be preferable for distinguishing virus taxa on different levels and in distinct virus groups. A plot of pairwise nucleotide and amino acid distances (Figure 3A) showed that all pairs of viruses formed two clearly distinguishable clouds in the rep gene. Out of a total of 5253 virus pairs, 3558 pairs differed by up to 7.5% nucleotide and up to 8% amino acid sequence, whereas 1695 virus pairs were different in more than 11% nucleotide and 6% amino acid positions. An artificial threshold of 10% nt could be thus suggested for robust AAV subdivision within the species level. At a protein sequence level, there was an overlap between distances within the four phylogenetic groups (below 7.5%) and between them (above 5.5%). Thus, the amino acid sequence is less suitable for the assignment of *rep* genotypes. However, no conclusions could be made according to the plot of pairwise nucleotide and amino acid distances of the cap gene (Figure 3B). The robust separation between AAV4, AAV11, AAV12 and all other serotypes (pairs differing in above 32.5% amino acids in 2250–4400 nt genomic region) was caused by a recombination event that led to the fact that fragments of AAV4, AAV11 and AAV12 capsids have an independent origin from AAV1, AAV2, AAV3, AAV6, AAV7, AAV8, AAV10, AAV13 capsids (Figure 3C).

Analysis of the individual AAV pairs in both plot areas showed that some of them are currently registered as distinct serotypes, but differ by less than 10% of the rep nucleotide sequence and thus may be assigned to the same *rep* genotype (Table 1).

**Figure 4:**
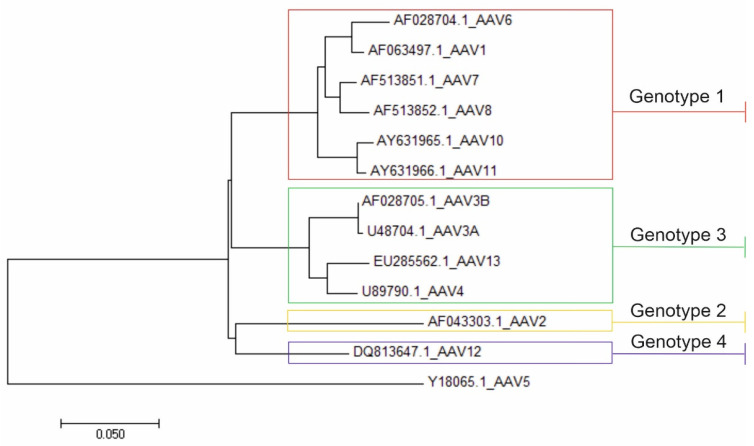
Neighbor-joining phylogenetic tree for the 453–1700 nt region of the AAV A genome. Correlations of currently registered serotypes and four suggested genotypes are presented. AAV B represents an outgroup.

There were no virus pairs in the dataset that were assigned to the same serotype, but differed by more than 10% nucleotide sequence in the rep gene. According to this concept, four distinct rep genotypes could be identified (Table 2, Figure 4).

## 4. Discussion

The lack of an established system for AAV subdivision below the species level results in uncertain classification. There is also no clear, generally accepted definition of such terms as “strain”, “variant” and “isolate”, and most publications simply reproduce the terminology used previously [46]. The currently used term, “serotype”, is not a classical taxonomic category, although such intraspecific division is applicable for practical and scientific purposes [47,48,49].

According to published studies, the historically established AAV classification based on the serotypes was in some cases not appropriately supported by either serological or genomic data. During early genomic studies, several regions with significant differences between the AAV serotypes were identified within the VP1 sequence, with four domains containing unique sequences for each serotype. These regions were assumed to have a role in serotype-specific functions, such as antigen-antibody or cell receptor binding, which presumably also corresponds to different tissue specificity [33,50,51]. However, the relationship between the serotype and tropism is not clear-cut, because several serotypes efficiently infect similar tissues via both the same and different receptors, implying that capsid proteins may not be the only determinants of tissue tropism.

In subsequent studies, when assigning new AAVs into distinct serotypes, genetic differences (primarily in the capsid proteins) were used. However, conflicts and incongruences in AAV serotype assignment have been accumulating (see above). They were further complicated by serological cross-reactivity [14,50]. Uncertain serological distinction was reported not just for AAV1 and AAV6 [34,36,52], but also for AAV1 and AAV5 [35]. Cross-neutralization between AAV11 and AAV4 was also noted [36]. Many inconsistencies in AAV classification that historically relied on a mix of phenotypic and genetic data could be explained by natural recombination.

Recombination is a physical interaction of viral genomes that leads to gene combinations not existing in any parent. Natural recombination has been described in all known viruses. In the case of some, it may be relatively rare, but in many viruses, it is a regular occurrence that shapes the genetics of taxa. Recombination is commonly seen as a typical feature of RNA viruses, and distinct genome regions of a virus often have different recombination profiles [51,53]; however, small DNA viruses [52,54,55,56,57] and adenoviruses [58] also commonly recombine, which may complicate their taxonomic assignment. For instance, recombination within the VP1 sequences was shown for another ssDNA virus—human parvovirus B19 [59]. Genetic analysis of canine parvovirus (CPV) VP1 indicated that recombinants emerged from CPV-2 and CPV-2a or CPV-2b viruses [60]. It was also found that human bocavirus HBoV3 is a recombinant of HBoV and HBoV4 with a recombination breakpoint located near the VP1 start codon [61], and a recombinant of HBoV3 and HBoV4 was further revealed [62]. Apparently, this was also true in the case of AAVs.

Analysis of the genetic sequences of several AAV clones isolated from various primates showed recombination signs in cap genes [57]. The recombination events were also observed in some clones associated with AAV2 and AAV3 [35]. Genetic mosaicism of VP genes of AAVs was shown to be a result of recombination events [62]. Furthermore, the AAV6 genome was shown to be the result of AAV2 and AAV1 recombination [63].

Our analysis confirmed that natural recombination in AAV is ubiquitous and needs to be considered for any taxonomic implications. The effect of recombination on taxonomy may both be negative, when it shuffles genomes and precludes a definitive phylogeny and sequence distance criteria, and positive, when it mixes genes within, but not between, taxa, as seen in enteroviruses [53] or adenoviruses [58], for example. It is likely that the same phenomenon, the shuffling of rep genes within the suggested genotypes, but not between them, explains a very robust separation of within- and between-genotype genetic distances. This hypothesis is supported by phylogenetic analysis (Figure 2E) and many incongruent pairwise genetic distances within genotypes (under 10%, Figure 2C). When it comes to recombination (frequently within the rep genotypes, but never between them), the AAV genotype acts similar to enterovirus [64] and adenovirus species [60].

The difference between the suggested AAV *rep* genotypes is objective, measurable, robust, and unambiguous. To assign viruses to a particular genotype, their genome needs to be sequenced and the sequence has to be analyzed with phylogenetic methods. The same strategy has been suggested and successfully implemented previously for adenovirus classification [65].

Genetic analysis allowed for robust segregation of AAV into *rep* genotypes. However, the biological implications of such a classification remain largely unknown, as most AAV properties have been associated with the capsid. Thus, the suggested classification is not intended to replace the classical VP1-based one, but to supplement it, and further studies are required to assay its phenotypic implications. In other viruses, it is not uncommon to have different classifications for distinct genome regions. One example is rotaviruses that have a segmented genome and feature ubiquitous reassortment [66]. However, there are also examples in viruses with non-segmented genomes, when distinct recombination profiles in different genome regions shape taxonomic units linked with structural proteins and with non-structural (replication-associated) ones. Examples include both RNA viruses, such as picornaviruses [66], and DNA viruses, such as adenoviruses [58]. In these examples, there is a fixed set of capsids (corresponding to types) that are compatible with a variety of non-structural genes within a species. Each species has a defined set of types, and structural genes from different species are not compatible. In AAVs, however, we did not observe obvious limitations on recombination between various rep and cap genes. This is concordant with the possibility of the so-called pseudotyped recombinant AAV vectors, i.e., a genome of one serotype used as a cassette for a therapeutic gene of interest, while the other serotype genome is used to supply *rep* and *cap*. Satisfactory transfection and expression were shown for AAV2/4, AAV2/5, AAV2/6, AAV2/8 and AAV2/9 constructs [17].

Knowledge about AAV genetic diversity remains fragmentary. For instance, the complete genome or rep gene of AAV9 has not been sequenced yet (the only record available in GenBank refers to the VP1 complete coding sequence). Thus, our dataset lacks this serotype for analysis (conducted based on Rep coding sequence) and we could not assign AAV9 to any of the determined genotypes. It is likely that the robust rep genotype criteria suggested here would need to be refined in the future. Additionally, although the suggested classification is robust and is explained by an obvious biological phenomenon (recombination), its practical implications require further study.

## 5. Conclusions

Based on genome analysis, we have identified four distinct *rep* genotypes, which are proposed as an auxiliary classification within the AAV A species: AAV A genotype 1 (including serotypes AAV1, AAV6, AAV7, AAV8, AAV10, AAV11); AAV A genotype 2 (including AAV2 and other unclassified AAVs that differ genetically between each other by less than 10%—the majority of the currently known sequences); AAV A genotype 3 (including AAV3, AAV4, AAV13 and other unclassified AAVs that differ genetically by less than 10%); and AAV A genotype 4 (represented by AAV12).

## Figures and Tables

**Table 1:** Nucleotide sequence (453–1700 nt positions of full-genome sequence) differences (upper triangle) and inferred amino acid sequence differences (lower triangle) among reference genomes of the currently registered AAV serotypes. Each color indicates a distinct rep genotype based on the nucleotide sequence difference of >10% with representatives of other genotypes and <10% for genomes within a genotype. Nucleotide sequence differences values below 10% (0.10) are highlighted in bold.

	AAV1	AAV2	AAV3	AAV4	AAV6	AAV7	AAV8	AAV10	AAV11	AAV12	AAV13
AAV1	**0**	0.16	0.12	0.11	**0.02**	**0.03**	**0.04**	**0.05**	**0.05**	0.12	0.12
AAV2	0.17	**0**	0.14	0.14	0.15	0.15	0.16	0.16	0.15	0.14	0.14
AAV3	0.16	0.18	**0**	**0.05**	0.13	0.12	0.13	0.12	0.12	0.13	**0.05**
AAV4	0.14	0.19	0.06	**0**	0.12	0.12	0.13	0.12	0.12	0.13	**0.03**
AAV6	0.02	0.18	0.16	0.15	**0**	**0.05**	**0.06**	**0.07**	**0.06**	0.13	0.13
AAV7	0.03	0.16	0.16	0.15	0.04	**0**	**0.03**	**0.05**	**0.04**	0.12	0.12
AAV8	0.05	0.18	0.18	0.16	0.05	0.03	**0**	**0.05**	**0.05**	0.13	0.14
AAV10	0.07	0.17	0.16	0.16	0.08	0.07	0.07	**0**	**0.01**	0.13	0.13
AAV11	0.07	0.16	0.16	0.15	0.07	0.06	0.06	0.02	**0**	0.12	0.13
AAV12	0.15	0.19	0.19	0.17	0.15	0.14	0.16	0.16	0.16	**0**	0.13
AAV13	0.15	0.18	0.06	0.02	0.15	0.15	0.16	0.16	0.15	0.18	**0**

**Table 2:** Distinct AAV genotypes identified based on nucleotide sequence differences and the corresponding currently registered serotypes. The table shows the range of the proportions of different nucleotides between the respective genotypes.

	Genotype 1	Genotype 2	Genotype 3	Genotype 4
Genotype 1 (AAV1 + AAV6 + AAV7 + AAV8 + AAV10 + AAV11)	0	0.15–0.16	0.11–0.14	0.12–0.13
Genotype 2 (AAV 2)	-	0	0.14–0.15	0.14
Genotype 3 (AAV3 + AAV4 + AAV13)	-	-	0	0.13
Genotype 4 (AVV12)	-	-	-	0

## Data Availability

The data and source code presented in this study are openly available in the GitHub repository https://github.com/MarinaBeloukhova/AAVgenotypes (accessed on 21 April 2022).

## Supplementary Materials

The following are available online at https://www.mdpi.com/article/10.3390/v14051038/s1, Figure S1: Neighbor-joining phylogenetic tree for Dependoparvovirus genomic sequences (4000–5100 nt length, *n* = 272) retrieved from GenBank. AAV-A (*n* = 105) sequences were indicated by red color.
