# Supplementary figures and images for: Robust AAV Genotyping Based on Genetic Distances in Rep Gene That Are Maintained by Ubiquitous Recombination
Source: Viruses. 2022 May 13;14(5):1038. doi: 10.3390/v14051038 (PMC9143360; doi:10.3390/v14051038)

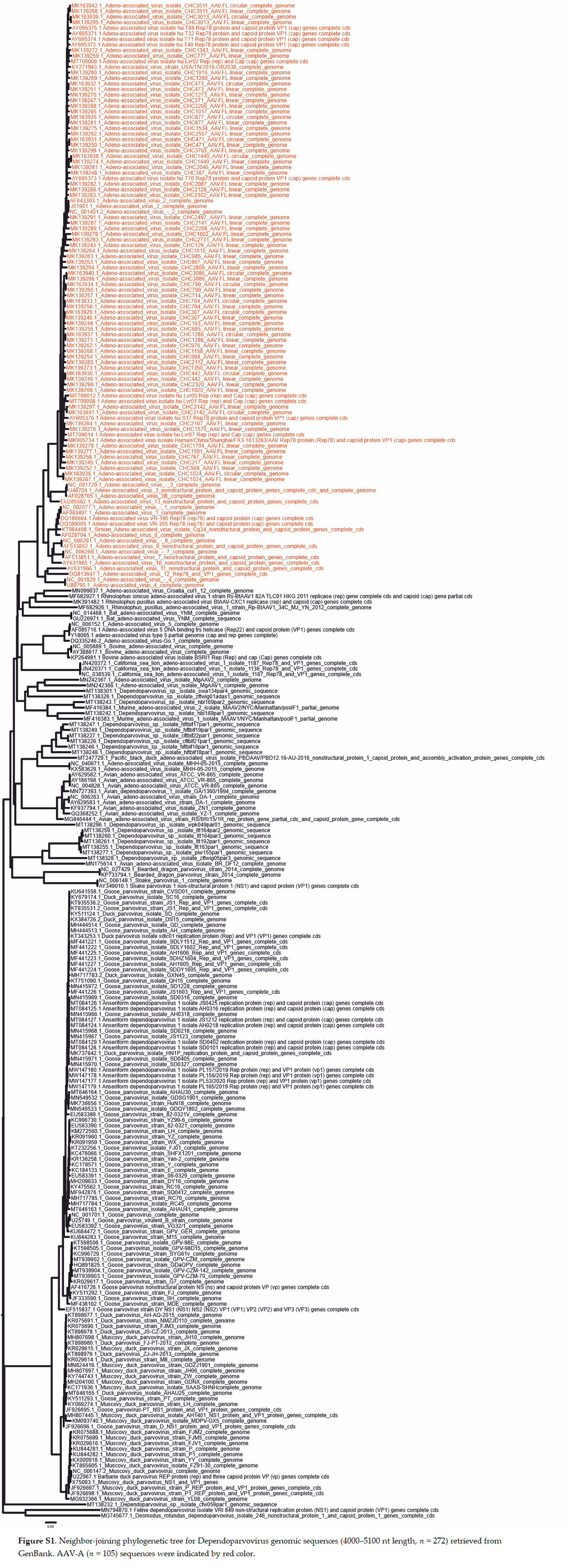

Supplement: Supplementary file 1 [file viruses-14-01038-s001.zip › Figure_S1.png]
